# Anticipating the downturn: business cycle forecasting for veterinary practice strategy in the United States

**DOI:** 10.3389/fvets.2025.1689704

**Published:** 2025-10-16

**Authors:** Clinton L. Neill, Matt Salois, Charlotte McKay

**Affiliations:** ^1^Department of Population Medicine and Diagnostic Sciences, College of Veterinary Medicine, Cornell University, Ithaca, NY, United States; ^2^Applied Economics Consulting, LLC, Blacksburg, VA, United States; ^3^Veterinary Study Groups Inc, Johns Creek, GA, United States

**Keywords:** veterinary economics, business cycle, forecasting, recession, practice management strategies

## Abstract

**Introduction:**

Veterinary medicine is often considered recession-resistant, yet little empirical analysis has evaluated the industry’s distinct economic cycle. This study addresses this gap by modeling and forecasting the veterinary medicine business cycle.

**Methods:**

Time series econometric techniques were applied to inflation-adjusted consumer price index (CPI) and expenditure data from 2000 to 2025. Dynamic autoregressive integrated moving average (ARIMA) models, incorporating macroeconomic indicators—industrial production, real disposable income, and consumer sentiment—were used to estimate and forecast monthly trends in CPI and real expenditures.

**Results:**

Forecasts reveal a continuing increase in veterinary service prices but a deceleration in real expenditures, indicating the industry entered a recessionary phase in late 2024. Prediction intervals suggest persistent negative growth through mid-2026, though with a potential for recovery toward the end of the forecast horizon.

**Discussion:**

These results demonstrate an industry-specific business cycle that does not necessarily mirror the macroeconomy. The veterinary industry’s current downturn presents both operational risks and strategic opportunities for practices, particularly in cost containment, workforce planning, and service innovation.

## Introduction

The dynamics of the veterinary medicine economy, namely industry-specific inflation and expenditure, experiences cycles of peaks and troughs like the larger business cycle of the macroeconomy. The business cycle is the recurrent pattern of growth and contraction in an economy usually represented by increases and decreases in aggregate expenditure, respectively. However, these cycles do not necessarily mimic those of the larger economy. This is due to the nature of the veterinary industry, along with external influences. The industry was dubbed as “recession-proof” or “recession-resistant” (both terms referring to having little to no decline in aggregate expenditure during macroeconomic recessionary periods) in a number of articles before and during the distinct shifts in veterinary service demand of the COVID-19 pandemic in contrast to macroeconomic declines (1–6). What is more likely is that the veterinary industry experiences a distinct, though not completely separated, business cycle that requires a deeper analysis.

As noted in previous research, the veterinary medicine industry is often linked to general consumer disposable income (7, 8) and willingness to adopt pets (9). While changes in the food animal sector are also important, in terms of labor more than two-thirds of veterinarians in the United States are linked to companion animal medicine (10). In addition, some research has suggested that the industry lags behind the larger economy as far as 7 years in terms of business cycle phases (11). Given these distinct differences and clear factors that influence the industry, better defining the business cycles for the veterinary medicine industry can provide current and future insight to manage risk at the practice level.

This article provides a framework to model the business cycle using time series econometrics along with forecasting. From this model and forecast, we contextualize the results to identify the phase of the business cycle the veterinary medicine industry is currently in and where it is headed in the next year based on this approach. In addition, management and business advice is then derived based on the current and future phase of the business cycle. While this model requires consistent updating every month, we have found our approach is robust from month to month. More importantly, the proposed framework can be utilized as a way forward to analyzing the industry’s cycles and provides a tool to be used in economic forecasting. Our specific research objectives for this article are as follows: (1) Model the veterinary business cycle using time-series econometrics; (2) Forecast sector-specific expenditures; (3) Define clear management strategies for individual practices during each business cycle phase.

## Materials and methods

### Materials/data

To examine the business cycles specific to the industry, data about the aggregate industry is needed such as inflation and expenditure. Veterinary Services consumer price index data is collected and reported by the United States Bureau of Labor Statistics (12). Aggregated expenditure on veterinary services throughout the United States is reported by the Bureau of Economic Analysis (13). Both data series are reported monthly. Additionally, we consider other macroeconomic variables that could influence both data series over time. We consider the impact of the following variables based on prior literature: Industrial Production (14) as a proxy for macroeconomic activity, University of Michigan’s consumer sentiment measure (15) to account for consumer expectations, and Real Disposable Income (16) to allow for changes in consumer income and costs. Industrial production is a common measure of macroeconomic activity and allows for us to include the larger economy movements into how they impact the veterinary sector. Consumer expectations is a good indicator of how people feel about the economy and if they plan to spend more in the coming months or in the current time period. Real disposable income, as noted before, is directly correlated with pet spending as this is the set of income that is often used to pay for veterinary services. Other variables were considered but found to have little to no impact on the fit of the econometric models. Factors such as pet abandonment, while important, lack reliable longitudinal data over the time period we model and were excluded for this reason.

**Figure 1:**
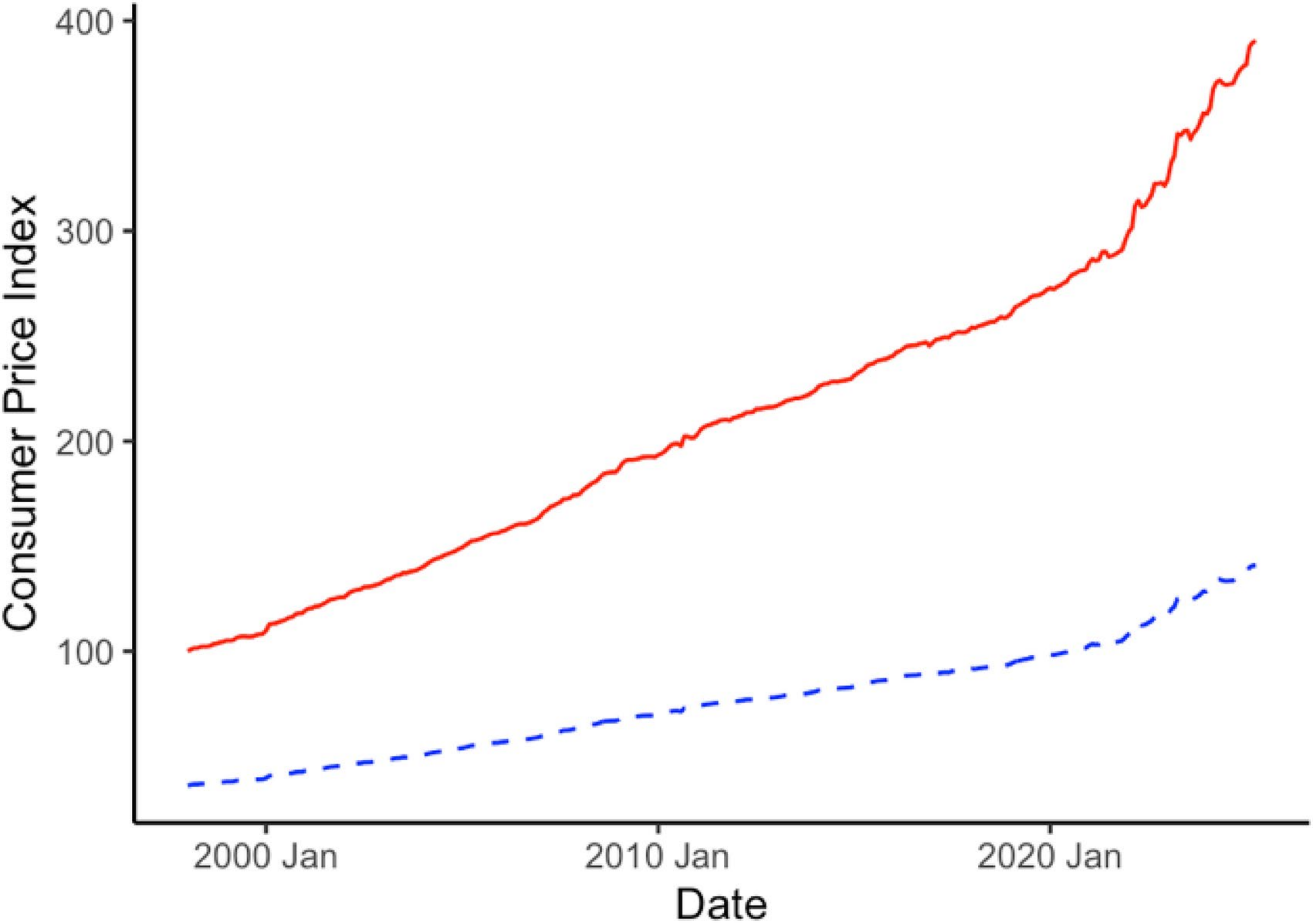
Veterinarian services consumer price index in original, 1997 dollar (red, solid line) and 2020 dollar (blue, dashed line) base values from the United States Bureau of Labor Statistics.

**Figure 2:**
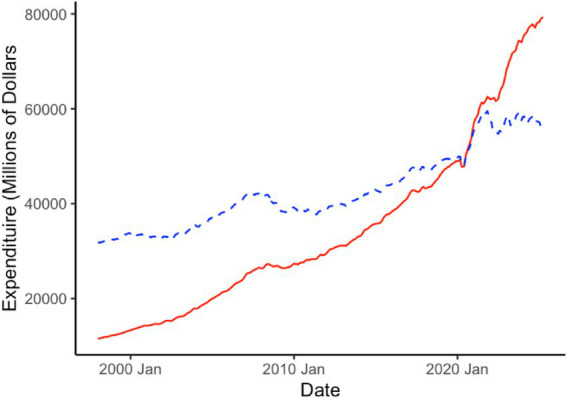
Veterinary service consumer expenditure in original, 1997 dollar (red, solid line) and 2020 dollar (blue, dashed line) base values from the United States Bureau of Economic Analysis.

**Figure 3:**
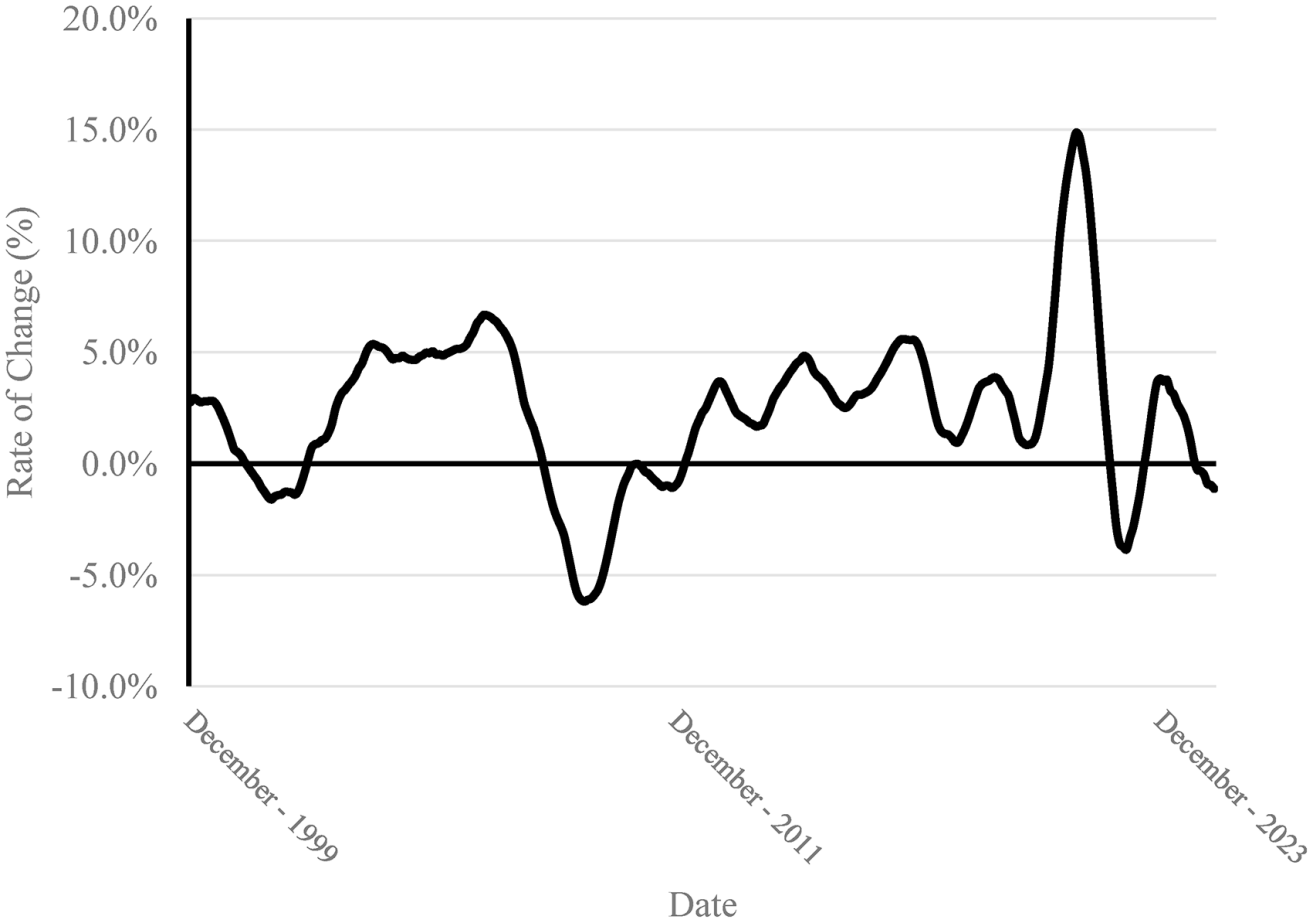
The 12 month, moving total rate of change of real, inflation adjusted (2020 dollar base value) of veterinary service expenditure. This is also denoted as the veterinary industry business cycle.

In order to characterize the business cycle for veterinary medicine, the industry specific consumer price index and expenditure are transformed to examine the real (inflation-adjusted) 12 month, monthly moving total rate of change of expenditure. By looking at the rate of change, this allows one to view how the industry is changing from year-to-year in total production/volume. We also change the base of the consumer price index from December 1997 to a 2020 average. This transformation does not affect model performance or the value of adjustment going from nominal to real dollar amounts. Instead, this allows us to interpret the results in terms of 2020 dollar amounts which isolates more recent changes in inflation brought on by the COVID-19 pandemic. In Figure 1, we present the original consumer price index (base = December 1997) and the adjusted consumer price index (base = 2020). In Figure 2, we present the nominal expenditure for veterinary services and the real, inflation-adjusted (base = 2020) expenditure series. Figure 3 shows the “business cycle,” also known as the 12 month, monthly moving total rate of change of real expenditure.

### Econometric methods—dynamic autoregressive integrated moving average model

In order to analyze how the business cycle moves, a dynamic autoregressive integrated moving average (ARIMA) model with seasonal components, where applicable, is used. The model is considered dynamic given we use external variables in addition to the data series themselves to determine how the series moves through time (17). We model the consumer price index and expenditure series separately rather than simultaneously. The general form of the consumer price index (CPI) dynamic ARIMA model is denoted as follows:


$${\text{ΔCPI}}_{t}=\beta_{0}+\beta^{'}X_{t}+\phi_{1}\eta_{t-1}+\varepsilon_{t}$$

where $\beta_{0}$ is the intercept term, β′X is the matrix of external variables (industrial production and consumer sentiment with their respective lag values) and the associated parameter values ( β ), $\phi_{1}\eta_{t-1}$ is the one time period lag autoregressive term, and $\varepsilon_{t}$ is the general regression error term. The general form of the expenditure (EXP) dynamic ARIMA model is denoted as follows:


$${\text{ΔEXP}}_{t}=\alpha_{0}+\alpha \prime Z_{t}+\phi_{1}\eta_{t-1}+\phi_{2}\eta_{t-2}+\theta_{1}\eta_{t-12}+\varepsilon_{t}$$

where $\alpha_{0}$ is the intercept term, $\alpha \prime Z_{t}$ is the matrix of external variables (industrial production, real disposable income, and consumer sentiment with their respective lag values) and the associated parameter values ( α ), $\phi_{1}\eta_{t-1}$ and $\phi_{2}\eta_{t-2}$ are the one and two period lags of the autoregressive term, $\theta_{1}\eta_{t-12}$ is the seasonal autoregressive term at a lag of 12 months, and $\varepsilon_{t}$ is the general regression error term.

Model selection is done by focusing on two criteria: minimization of the Bayesian Information Criterion and aiming for parsimony. The dynamic regressions are estimated using maximum-likelihood estimation (17). The specific external variables used in the consumer price index equation are lagged values of industrial production and lagged values of consumer sentiment. For the expenditure equation, those same external variables are used along with lagged values of real disposable income.

After estimating each of the models, we forecast each series for the next 12 months and then construct the business cycle measure. Due to some of the external variables being contemporaneous with the forecasts, an ARIMA model and forecast is conducted for each of those series as well. These values are then used to estimate the next 12 monthly values. Given these are predicted values there is uncertainty around our estimates. Therefore, we also estimate the upper and lower forecast prediction intervals at the 80% and 95% confidence levels using bootstrapping. Bayesian Information Criterion and log-likelihood values are used to evaluate goodness of fit of the model. The consumer price index, expenditure, forecast, and prediction interval series are then transformed to form the actual and forecasted business cycle series.

The industry specific business cycle, along with the forecasts, is an informative tool to indicate how veterinary practices can better manage the risks associated with broader changes in the industry and macroeconomy. Specifically, from the business cycle series, one can determine the phase of the business cycle the industry is in and where it is headed over the next year. We divide the business cycle into four phases based on movements in the rate of change in real expenditure: Recovery, Expansion, Contraction, and Recession. Below we detail the characteristic features of each phase based on the economic series used in this study.

### Recovery

In this early stage of the cycle, veterinary activity remains below the level seen 1 year prior; however, the year-over-year growth rate (rate-of-change) is beginning to climb. Despite general pessimism in the marketplace, this phase presents a strategic opportunity. Practices that recognize the shift in momentum can benefit from investing in infrastructure, equipment, or undervalued assets before broader market optimism returns (18). Initiatives in marketing or community engagement launched during this time can build early momentum. It is also essential to assess staffing and capacity needs to ensure readiness for the forthcoming period of accelerated growth (19).

### Expansion

Expansion represents the strongest segment of the cycle, with the rate-of-change not only positive but also gaining speed. Veterinary demand is accelerating, and practices should capitalize on this momentum by strengthening client relationships, expanding service offerings, and reinforcing team development (20). Hiring and continuing education initiatives are particularly beneficial in this phase (21). For practice owners considering a transition or sale, this period of strong economic confidence may offer the most favorable conditions for maximizing valuation (22).

### Contraction

Although veterinary activity may still show positive growth compared to the previous year, the growth rate is slowing. This phase can be deceptively optimistic, as headline indicators remain strong even as momentum begins to wane. Caution is warranted as practices should prioritize liquidity, limit unnecessary spending, and avoid new long-term obligations that may not be sustainable if conditions deteriorate (23). Financial discipline during this phase, especially in areas like inventory management, capital expenditures, and staffing, can help mitigate the risks of an impending downturn (24, 25).

### Recession

In this stage, both economic activity and the rate-of-change are in decline. While this environment presents challenges, it also allows practices to reassess operations and prepare for the next growth phase. Cost containment becomes a priority—adjustments to marketing, credit policies, and staffing strategies may be necessary (26, 27). Leadership is especially critical during recessionary periods (28); maintaining transparent and positive communication can help preserve team morale. For younger staff unfamiliar with economic downturns, managerial composure can provide stability and direction (29). Practices that act decisively on early recovery signals will be best positioned to thrive as the cycle resets (30, 31).

## Results

**Figure 4:**
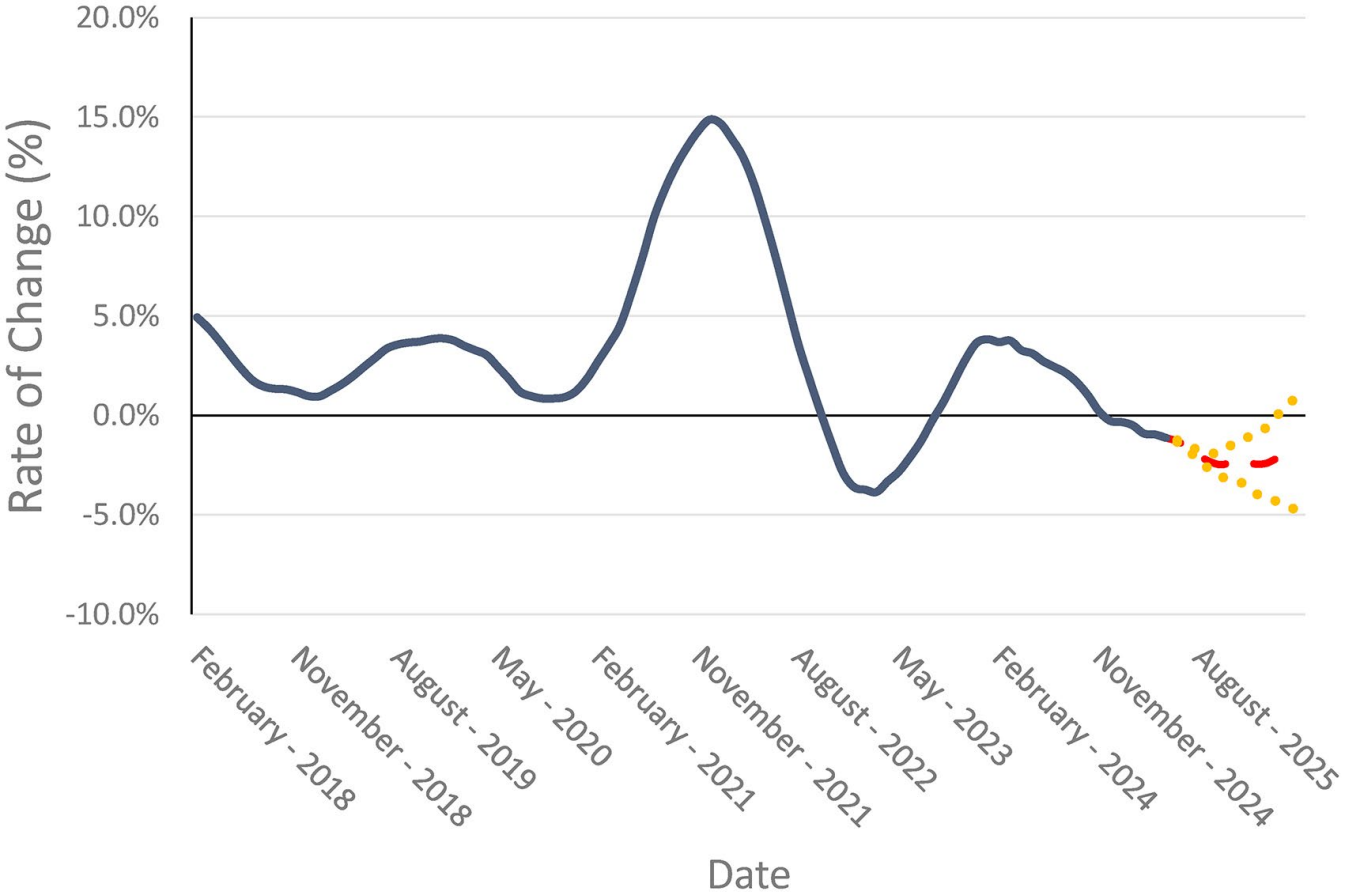
Forecasted values of the veterinary business cycle (12 month, moving total rate of change of real, inflation adjusted (2020 dollar base value) of veterinary service expenditure) for the next 12 months (June 2025 through June 2026). Forecasted mean values are represented by dashed red line, 95% prediction intervals are denoted by the dotted yellow lines.

The resulting forecast for the business cycle (12 month, moving total rate of change) is presented in Figure 4. The dynamic ARIMA model has smaller prediction intervals than an ARIMA only model, especially for the near-term forecasts. The forecasts for each series, the prediction intervals, and the goodness-of-fit measures are presented in Table 1.

**Table 1:** Forecasted values and goodness of fit measures for veterinarian services consumer price index and veterinary services expenditure time series.

Month	Veterinarian services consumer price index (base = 2020)				
	Forecasted values	80% LPI	80% UPI	95% LPI	95% UPI
June 2025	141.32	141.00	141.67	140.56	142.57
July 2025	141.64	141.02	142.42	140.35	144.21
August 2025	141.93	141.03	143.05	140.24	145.09
September 2025	142.26	141.11	143.67	140.30	145.71
October 2025	142.58	141.23	144.33	140.38	146.27
November 2025	142.92	141.35	144.98	140.51	146.82
December 2025	143.25	141.52	145.55	140.64	147.36
January 2026	143.58	141.68	146.06	140.73	147.89
February 2026	143.91	141.89	146.55	140.81	148.48
March 2026	144.25	142.09	147.01	140.90	148.96
April 2026	144.58	142.28	147.43	141.05	149.59
May 2026	144.88	142.45	147.93	141.25	150.07
BIC	504.52				
Log-likelihood	237.79				

Month	Veterinary services expenditure (base = 1997)				
	Forecasted values	80% LPI	80% UPI	95% LPI	95% UPI
June 2025	$ 79,333.57	$ 79,108.01	$ 79,557.49	$ 78,953.12	$ 79,961.46
July 2025	$ 79,466.18	$ 79,044.25	$ 79,927.71	$ 78,737.68	$ 80,641.13
August 2025	$ 79,638.76	$ 78,990.24	$ 80,378.49	$ 78,591.42	$ 81,254.10
September 2025	$ 79,807.97	$ 78,936.47	$ 80,793.41	$ 78,443.28	$ 81,904.08
October 2025	$ 80,145.60	$ 79,036.57	$ 81,407.85	$ 78,484.58	$ 82,678.09
November 2025	$ 80,528.07	$ 79,203.89	$ 82,052.95	$ 78,556.72	$ 83,470.80
December 2025	$ 80,532.93	$ 79,008.94	$ 82,278.48	$ 78,257.79	$ 83,839.30
January 2026	$ 80,733.72	$ 79,017.96	$ 82,701.60	$ 78,173.44	$ 84,516.91
February 2026	$ 81,001.16	$ 79,080.51	$ 83,210.14	$ 78,148.35	$ 85,170.42
March 2026	$ 81,106.11	$ 79,011.64	$ 83,482.69	$ 78,035.50	$ 85,645.18
April 2026	$ 81,291.73	$ 79,027.78	$ 83,836.82	$ 77,972.83	$ 86,100.99
May 2026	$ 81,534.16	$ 79,083.83	$ 84,291.31	$ 78,064.89	$ 86,520.91
BIC	4432.23				
Log-likelihood	2190.06				

LPI, lower prediction interval; UPI, upper prediction interval at the respective confidence level; BIC, Bayesian information criterion from the respective dynamic model.

Veterinary services consumer price index has grown consistently over time since 2000, much like overall inflation. Note that here we refer to it as “veterinary” services consumer price index, though it is officially called “veterinarian” services by the Bureau of Labor Statistics. However, the rate of growth has ebbed and flowed at different times—much of which can be attributed to the larger macroeconomy. Since the pandemic, veterinary service inflation has been higher than economy-wide inflation during the observed time period other than the 2001 macroeconomic recessionary period. Inflation for the industry experienced the lowest amount of inflation after the Great Recession until the Pandemic era. In the last 4 years, however, inflation has been well above the rest of the time series but has been declining over the last year. This is reflected in the rate of change trend and shows that the veterinary industry level inflation is cooling.

Veterinary service expenditure has been more cyclical since 2000 and has generally followed a larger macroeconomic trend. Over the long term, expenditure has been growing with some decline after the Great Recession and at the onset of the Pandemic. The recovery in expenditure was rapid after the initial Pandemic decline and continued a strong pattern of growth until late 2024. In the last year, veterinary service expenditure has generally been on the decline.

From the dynamic regression forecast of the business cycle, it is apparent that the recent trends in both consumer price index and expenditure are expected to continue. The veterinary service consumer price index is forecasted to continue a mostly linear increase over the next 12 months. Nominal veterinary service expenditure is also expected to increase month-to-month over the same time period. However, when the series are combined to look at real expenditure, the impact of inflation creates a more cyclical effect on expenditure, with some short-term declines and a modest growth over the forecasted period. The prediction intervals for both series are small at the beginning of the forecasted time period and grow the further away from the current time period due to increased uncertainty. In-sample and out-of-sample forecast robustness checks are within the 80% confidence range, indicating close to accurate forecast.

With the forecasts for each series, the business cycle (12 month moving total rate of change) series is constructed to determine the past, current, and forecasted phases of the veterinary medicine economy. The business cycle shows that the veterinary medicine economy had past periods of recession that corresponded, if slightly lagged, the macroeconomy recessions. A recession in this framework is denoted as a period with negative changes in the business cycle series. From the forecast for the business cycle, the veterinary medicine economy appears to be in a recessionary period that began November 2024 and is expected to extend well into next year with every month in the forecast showing negative growth. The prediction intervals for the end of the forecast period do indicate much more uncertainty leading to a chance that the veterinary economy could recover and be in the positive growth territory around the second quarter of 2026. It is important to note that this forecast assumes ceterus paribus, which means that “all else equal” and no shocks or structural shifts occur in the future. The forecasts are estimates with broad confidence intervals (95%) and should be updated every month based on the most recent data.

## Discussion

As noted earlier, the business cycle series and forecasts indicate that the industry is in a negative growth phase which is denoted as a recession. Practice level management strategies can be devised that manage the risk of loss and prepare for the next phase of the industry specific economy. Each phase of the business cycle requires different strategies. Below we detail management strategies that have proven useful for each phase denoted earlier. It is important to note that each individual practice needs to consider what works for them, as not all will be appropriate, especially depending on how local market dynamics differ from national macroeconomic trends. However, the general idea of managing cash flows, investments, and labor is key in each set of recommendations. Ultimately, aligning strategic management decisions with the current phase of the business cycle can help practices maximize opportunity during periods of growth and minimize risk during downturns.

### Recovery

The recovery phase presents a pivotal opportunity for veterinary practices to reposition themselves for sustainable growth. During this period, it is critical to rigorously assess the reliability, efficiency, and cost-effectiveness of the supply chain. This includes evaluating relationships with pharmaceutical distributors, lab service providers, and consumable vendors to ensure pricing, availability, and delivery terms are aligned with future needs (22, 23). Practices may consider diversifying suppliers for critical items like vaccines, surgical supplies, and diagnostic equipment to reduce risk.

Leadership behavior should actively model optimism and accountability, as organizational culture during recovery is especially impressionable and will guide future operational norms^26^. Practices should begin phasing out underperforming products, services, and personnel to shore up profit margins and enhance resource allocation (23). Team morale during recovery is especially impressionable, and leadership tone will shape the cultural foundation for the next phase of growth (29). Initiatives such as regular staff huddles, recognition programs, and open Q&A forums can foster resilience and engagement. Strategically, this is a critical time to phase out underperforming services, products, or personnel. For example, discontinue niche services that consistently operate at a loss or repurpose underutilized equipment to support more profitable care areas. Similarly, personnel reviews should identify individuals misaligned with the clinic’s culture or strategic direction, and difficult decisions about staffing may be necessary to ensure long-term viability (30).

Financial flexibility is key in recovery. Practices should also explore selective credit extensions and offer structured payment plans for loyal clients undergoing economic hardship (27). Simultaneously, re-engage financial partners—banks, leasing companies, or private lenders—to secure access to capital before expansion peaks (28). Lines of credit, equipment loans, or SBA financing can be lined up now while balance sheets are improving and interest rates may be favorable (27, 28).

Client market research is essential. Short surveys, post-visit feedback, or focus groups can uncover changing preferences—for example, an increased interest in convenience services like telemedicine, subscription wellness plans, or extended hours (10, 22). These insights should inform pricing strategies, staffing models, and service offerings. Hiring and training should begin proactively, even before caseloads fully return to peak (22, 26). Recruiting credentialed veterinary technicians, CSRs with strong client communication skills, and future leaders in middle management ensures the team is ready for the demands of expansion (11, 22). Implementing a structured onboarding and training program—covering both medical protocols and client service expectations—builds confidence and consistency (22).

Marketing and sales efforts should be scaled up in alignment with growing veterinary demand. Reactivate digital advertising, launch client re-engagement campaigns (e.g., “We Miss You” wellness reminders), and spotlight new or returning services. Pair this with strategic investments in workflow and system efficiency (30) such as:

- Implementation of online appointment scheduling and reminders to improve client convenience and reduce phone traffic (22);
- Utilizing advanced practice management software, especially cloud-based ones, that supports real-time analytics, client communication, and inventory tracking (23);
- Adoption of AI tools for radiology interpretation, treatment plan generation, or client education to streamline clinical operations (22);
- Use of automated inventory management systems that reduce waste and enhance cost control by tracking product use in real time (22, 23).

Finally, veterinary leaders should remain alert for opportunistic acquisitions, such as buying equipment from closing clinics, taking over leaseholds, or acquiring a retiring veterinarian’s client list. With some market participants still cautious or retreating, the recovery phase can provide excellent opportunities to acquire strategic assets at favorable terms and position for long-term competitive advantage (31).

### Expansion

As the economy and industry conditions improve, the growth phase demands disciplined execution to ensure that the quality of care and operational performance scale in tandem with rising client demand. Veterinary practices must implement and reinforce standard operating procedures (SOPs) for all clinical and client workflows. These SOPs should be reviewed and updated regularly to reflect current best practices in areas such as anesthesia protocols, infection control, patient handoffs, and exam room communication^21^.

Maintaining quality control during high-volume periods also requires an intentional focus on ongoing staff training (11). Practices should introduce competency checklists, role-based onboarding tracks, and monthly in-clinic CE sessions (22). Consider assigning team leads for departments (surgery, dental, wellness, client service) who can monitor adherence to protocols and identify training needs early.

Up-to-date equipment is another vital element of quality scaling (22). Growth-phase practices should invest in tools that improve clinical accuracy and efficiency—such as dental radiography, cold laser therapy units, ultrasound, or cloud-connected lab analyzers. These not only enhance care but can be marketed as differentiators to clients.

Workforce development remains a cornerstone of successful growth (26). This means:

- Strategic hiring of technicians, doctors, and support staff before bottlenecks emerge (22);
- Offering retention incentives—such as tiered bonuses, growth tracks, or leadership pathways for top performers (26);
- Utilizing float positions or per diem staff to manage peak periods and reduce burnout (11).

Pricing strategy should be actively managed. Practices should:

- Review their fee schedules at least annual (22);
- Include cost escalators tied to inflation of labor cost benchmarks (12, 16);
- Communicate any price increases clearly and with empathy, emphasizing the value of services rather than just the price (29).

Differentiation becomes increasingly important at this stage with a crowded marketplace. Practices should seek to strengthen their unique value proposition, distinguishing themselves in a competitive environment to maximize margins (28). This could include fear-free certification and low stress handling, same-day urgent care availability, extended evening or weekend hours, or specialized services like exotic pet medicine, geriatric wellness, or acupuncture.

Increased cash flow in this phases should be used to build resilience, not overextend. This could include:

- Creating a cash reserve to weather future slowdowns (18);
- Investing in scalable infrastructure like upgraded software, cloud storage, or cybersecurity protections (23);
- Conducting a risk audit to identify operational vulnerabilities before they are stress-tested by future downturns (31).

Expanding client credit offerings can drive additional business, provided these are managed with sound risk assessment. Some examples include:

- In-house payment places for procedures over a certain threshold (27);
- Partnering with third-party financing services (e.g., CareCredit) (27);
- Offering subscription-style wellness plans that promote preventive care and client retention (10).

Improved corporate governance should also be a focus to support long-term sustainability. Some examples include:

- Hiring part-time financial leadership like an accountant or controller to advise on cash flow, tax strategy, and capital planning (23);
- Establishing an advisory board of external professionals (e.g., veterinary consultants, financial advisors, legal counsel). to formalize oversight structures (31);
- Using financial dashboards to monitor KPIs like cost of goods sold, revenue per doctor, client churn, and average transaction charge (10).

Marketing efforts should pivot towards clear client communication of the practice’s competitive advantages and reinforcing brand values. This includes:

- Highlighting staff credentials and new service offerings through social media (22);
- Launching Google review campaigns and referral incentives (29);
- Showcasing community involvement (e.g., adoption events, wellness seminars, partnerships with shelters) (22, 29).

Additionally, practices should implement continuous client feedback loops via surveys, text follow-ups, or QR code review requests at check-out (10). These mechanisms help align services with evolving client needs and can provide actionable insights into service satisfaction, emerging needs, and pricing sensitivity (22).

For some practice owners considering a transition, this growth phase is often an ideal time to prepare for a potential exit via sale or partner buy-in. With strong financials, positive market sentiment, and peak goodwill, valuation potential is at its highest. Owners should begin assembling clean financials, evaluating buy-side interest, and working with brokers or consultants if a sale is part of the strategic horizon (31).

### Contraction

Veterinary practices often fail to recognize the onset of a contraction phase until the symptoms—slower appointment growth, reduced foot traffic, or lower prescription sales—already taken root. To mitigate this, proactive monitoring of rate of change metrics—such as monthly revenue, number of active clients, transaction counts, and average transaction value—can offer an early warning that the business has peaked and must transition to more conservative management. Dashboards and simple 12/12 trend comparisons can provide early signals that growth is decelerating, even if headline numbers remain positive.

During contraction, practices should begin revisiting their capital expenditure plans, preserving cash as a protective measure (26). Delay or cancel major investments in non-essential renovations, new equipment, or technology unless they are mission-critical or immediately ROI-positive (24). Instead, preserve cash to improve short-term liquidity and maintain operational flexibility.

Practices should conduct a margin analysis of all services and products to identify underperforming offerings (24). Low-performing services and product lines should be discontinued in favor of higher-margin offerings, while operational costs should be reassessed with a focus on renegotiating vendor contracts and securing more competitive pricing (26). For example, consider discontinuing low-utilization elective services like laser therapy or non-core inventory SKUs with slow turnover (26). Another example may involve shifting focus to high-margin areas like dentistry, diagnostics, or preventive care packages that can be delivered efficiently with existing staff and infrastructure (10).

Although the practice may still be growing relative to the prior year, leadership must resist the temptation to maintain the status quo and instead adopt forward-looking tactics that preserve profitability (28). Understanding whether the market is heading for a soft (economic slowdown but no recession) or hard landing (large economic downturn often leading to a recession) is essential, and budgets should reflect the likely scenario without assuming linearity (18). Preparing for a “soft landing” budget involves modest growth with tighter margins. On the other hand, preparing for a “hard landing” involves expense cuts, reduced hours, or hiring freezes.

Vigilant management of accounts receivable is crucial to maintaining liquidity (25), and selective pricing strategies can help build a backlog that cushions future revenue dips. Some examples include:

- Implement tighter credit policies and shorter payment windows (27);
- Use automated reminders and payment links via text or email to accelerate collections (27);
- Offer early payment incentives for larger invoices (e.g., surgical packages) (28).

Pricing strategies should become more selective and strategic. Practices can:

- Use value-based bundling (e.g., preventive care bundles, vaccine packages) to maintain volume while avoiding individual price reductions (22);
- Introduce limited-time promotions to stimulate demand during soft periods (26);
- Maintain premium service tiers while adding budget-friendly alternatives (e.g., optional tech appointments, telehealth rechecks) (26).

Practices should avoid entering long-term expense commitments (26) at peak prices but seek opportunities to lock in favorable revenue contracts – this includes leases, equipment financing, and multi-year marketing contracts. However, this is also a smart time to lock in favorable revenue contracts where possible. For example, renew long-term wellness plan agreements before economic uncertainty deepens.

In more aggressive downturns, leadership may need to consider cross-training employees to maintain flexibility in staffing and ensure operational continuity by maintaining strong client relationships (21). Some examples include:

- Train client service reps in basic tech duties or inventory management (26);
- Equip veterinary assistants to float across exam room, pharmacy, and kennel operations (22);
- Ensure flexibility in scheduling and allow teams to absorb staffing gaps without disrupting care (26).

It is also important to continue to maintain strong client relationships, which will be imperative through a recession and into recovery. Provide transparent communication (21) about pricing, service value, and wellness plan benefits. Implement “Ask Me Anything” events or webinars to keep pet owners engaged. Use text and email updates to build loyalty and maintain appointment cadence. This is also the time to explore entrepreneurial or counter-cyclical service lines that may buffer against broader economic weakness^30^. Some examples include:

- In-house pharmacy delivery for convenience-oriented clients (22);
- Pet nutrition consulting bundled with retail sales of food and supplements (22);
- Behavioral health services as more pets experience stress during lifestyle changes at home (26).

By recognizing the signs of contraction early and taking disciplined action, veterinary practices can avoid crisis-mode responses and maintain profitability through the downturn. Even more importantly, they’ll be better positioned for a strong rebound when the next recovery begins.

### Recession

Though often viewed with apprehension, the recession phase (or trough of the business cycle) can be a strategic reset point for veterinary practices. Rather than responding with panic or retrenchment, practice owners should lead with measured optimism and a focus on preparing for the next cycle. This quieter period allows leadership to step back and assess the business holistically. Freed from the relentless pace of peak demand, owners can invest time in generating and evaluating ideas for new services, products, market segments, or even investment opportunities that were previously constrained by day-to-day demands. Some examples might include:

- Subscription-based wellness plans that emphasize preventive care and monthly affordability (10);
- Telemedicine services for follow-ups and minor concerns, which offer convenience while reducing overhead (22);
- Senior pet care packages or end-of-life planning—services that meet evolving client needs while strengthening the human-animal bond (26).

It also creates the space to internally assess departmental performance and streamline inefficiencies. Evaluate the profitability and utilization of surgery, dental, and wellness services. Assess scheduling efficiency, inventory turnover, and technician utilization ratios. Or, eliminate or consolidate duplicative or inefficient workflows to streamline operations.

Marketing and creative teams can focus on developing campaigns and content that will be deployed as the market rebounds, ensuring the practice is well-positioned once demand strengthens. Marketing and creative teams should shift focus from short-term promotions to building future-facing content. One approach is to develop evergreen blog posts, educational videos, and email campaigns to deploy during the recovery. Or, refresh your website messaging and client resources to better reflect your value proposition. It’s also an ideal time to identify new market segments (21), such as:

- Partnering with local shelters or rescues for ongoing care contracts (26);
- Creating loyalty or wellness programs specifically for multi-pet households (22);
- Offering “value visits”—short, technician-led appointments for specific concerns like nail trims, anal gland expression, or brief wellness checks (22).

From a financial standpoint, now is the time to implement aggressive cost-cutting measures, particularly through attrition of underperforming staff or realigning roles. Some options include: leaving open positions unfilled if they are not essential (26), restructuring schedules to reduce overtime or weekend premiums (31), or cross-training staff so fewer team members can cover more ground during slower days (31).

Introducing lower-cost product options may appeal to more price-sensitive clients, while also expanding the practice’s reach. For example, offer generic medications when appropriate and communicate savings to clients (22). Another option is to create budget-friendly service tiers (e.g., basic dental vs. full-mouth radiographs + cleaning). Or use bundled pricing (e.g., vaccine + wellness exam) to enhance perceived value while preserving margins (26).

New product development and market research initiatives (26) launched during this time can yield competitive advantages in the recovery phase by capturing client attention during a time when there is less market activity. Some things to consider include:

- Conduct client surveys about service priorities, price sensitivity, and unmet needs (22);
- Prototype and pilot new services on a small scale before full rollout (26);
- Rebrand and refresh marketing messages to better align with emerging client values (e.g., transparency, affordability, convenience) (31).

Inventory should be managed carefully. Avoid over-ordering slow-moving stock keeping units (SKUs) but monitor for critical supply availability and rebuild essential inventories—like vaccines, suture materials, and commonly prescribed meds—as signs of recovery emerge, ensuring short lead times when volume returns (23).

Advertising spend may be reduced temporarily, but contracts, leases, and vendor agreements should be renegotiated to lower long-term liabilities (23). For example, work with landlords to explore lease renegotiation or extensions at lower rates. If you are a member of buying-group or have access to a purchasing program through a member-based organization (e.g., VMG or PSI), make sure you are fully leveraging the savings through those agreements and their chosen partners (31).

Practices should evaluate credit policies (28) to ensure they reflect tighter risk tolerances and the increased risk of nonpayment. One tactic is shift to prepayment for certain procedures or new clients. Your practice should also look to reduce the maximum allowable outstanding balance per client. You can also offer financing through third-party providers instead of carrying client debt on your books.

Finally, evaluate and identify capital equipment needs in anticipation of the next recovery to support growth once the recovery begins (22). Make a list of aging equipment (e.g., autoclaves, anesthesia machines) and gather quotes now. Consider leasing options that allow for deferred payments or lower upfront capital outlay. Use any downtime to upgrade or service existing equipment without disrupting daily operations (23).

Even during economic downturns, well-prepared veterinary practices can reposition themselves for long-term growth and strength. By using this time during a recession phase to reposition themselves, align operations with long-term goals, sharpen their value proposition, and investing in resilience, practices can emerge stronger, more agile, efficient, and competitive when the cycle turns upward once again (31).

### Conclusion

While the veterinary industry is in a recessionary period that could last at least another 12 months (based on the business cycle series and forecasts), it is important to consider that there are ways to manage the risk of the economic trough. Managing risk is part of any business and there are connected markets within animal health (9) that will affect the near-term future of the industry. However, the industry has shown long-term growth, and it is important for practices to prepare for the recovery phase of the business cycle to capitalize on the next economic expansion. Practices can still realize profitable growth during the current recession period as well, which will continue to support current staff, clients, and patients. The latest forecasts using this method and other insights can be found at https://www.myvmg.com/knowledge-center/economic-dashboard/.

From a policy perspective, our results indicate that the veterinary sector is somewhat distinct from the larger economy but there are clear tethers. For example, when disposable income declines due to economy wide inflation or job loss, the veterinary sector is also expected to decline in economic activity. Policy measures around affordability and access could assist in making the veterinary sector more recession-resilient. Moreover, policymakers and industry associations should not rely solely on aggregate economic indicators to anticipate veterinary sector stress. Dedicated veterinary-specific monitoring could help regulators, trade groups, and insurers recognize downturns earlier and create policy interventions that assist practices, pet owners, and veterinary team members weather the veterinary economic cycle.

## Data Availability

The original contributions presented in the study are included in the article/supplementary material, further inquiries can be directed to the corresponding author.
